# Cost-effectiveness of ustekinumab in moderate to severe Crohn’s disease in Sweden

**DOI:** 10.1186/s12962-018-0114-y

**Published:** 2018-08-02

**Authors:** Amanda Hansson-Hedblom, Chrissy Almond, Fredrik Borgström, Indeg Sly, Dana Enkusson, Anders Troelsgaard Buchholt, Linda Karlsson

**Affiliations:** 1 Quantify Research AB, Stockholm, Sweden; 2 BresMed, Sheffield, UK; 3 Janssen-Cilag AB, Solna, Sweden

**Keywords:** Crohn’s disease, Ustekinumab, Adalimumab, Vedolizumab, Cost-effectiveness

## Abstract

**Background:**

Human monoclonal antibody ustekinumab is a novel Crohn’s disease (CD) treatment blocking pro-inflammatory cytokines interleukin-12 and 23. The study’s objective was to assess cost-effectiveness of ustekinumab in moderate to severely active CD in Sweden.

**Methods:**

A cost-effectiveness model with an induction phase decision-tree structure and a maintenance phase Markov cohort structure was constructed. CD was represented by five health-states: remission, mild, moderate-severe, surgery and death. Ustekinumab was compared to adalimumab in patients who had failed conventional care, some of which had tried TNF-alpha-inhibitor(s) without experiencing treatment failure or side effects (“conventional care failure population”) and to vedolizumab in patients previously failing TNF-alpha-inhibitor treatment. Discontinuation probabilities, utilities and ustekinumab induction efficacy were sourced from phase-III trials. Maintenance and comparator efficacy came from network-meta and treatment-sequence analyses. Resource use and unit costs were derived from literature and validated by clinical experts. The analysis had a societal perspective, a life-time time-horizon, and 2-year treatment duration. The results robustness was tested in univariate and probabilistic sensitivity analyses. Cost-effectiveness was estimated using quality-adjusted life-years (QALYs).

**Results:**

Ustekinumab dominated adalimumab in conventional care failure population (costs: − €6984, QALYs: + 0.232). In TNF-alpha-inhibitor failure population ustekinumab accrued 0.133 more QALYs than vedolizumab, yielding a €30,282 incremental cost-effectiveness ratio. Results were sensitive to decreasing the time horizon and increased treatment duration. At Swedish reference willingness-to-pay of €63,000 (SEK 600,000), ustekinumab had 94% probability of being cost-effective versus adalimumab, and 72% versus vedolizumab.

**Conclusions:**

Results indicate ustekinumab dominates adalimumab in conventional care failure population, and is cost-effective versus vedolizumab in TNF-alpha-inhibitor failure population.

## Background

Crohn’s disease (CD) is a relapsing–remitting inflammatory bowel disease which mainly affects the gastrointestinal tract. Common symptoms include abdominal pain, fever, bowel obstruction and diarrhoea with passage of blood or mucus, or both [1]. Disease complications include malnutrition, fistulas, fissures and abscesses [2]. Approximately 20,000 people in Sweden are affected by CD [3], with around 10 new cases per 100,000 person-years [4]. There are indications that CD incidence and prevalence are increasing [5–10] with most patients being diagnosed between the ages of 15 and 30 [3]. The disease is associated with significant treatment costs, productivity losses and a substantial quality of life impact [3, 11]. The annual per patient cost of CD in Europe has previously been estimated between €6024 and €22,581. The costs were related to primary and secondary care, medical and surgical hospitalization, and labour force non-participation [12–14]. A Polish study from 2016 estimated the annual indirect costs at around €5550 per patient [15]. The cost of CD is expected to increase due to the shift from hospitalisation and surgery towards biologic therapy use, shown in the COIN study [16]. Reported health related quality of life (HRQoL) is significantly lower in CD patients as compared to normal populations [12]. It has been estimated that more than 20% of Crohn’s disease patients will require surgery within 5 years of diagnosis, and approximately 40% within 10 years [17]. Common CD surgeries include strictureplasty, resection, and colectomy [18].

There is no cure for CD, but a variety of disease managing, often long-term, treatments are available [19]. Biological treatments including tumour-necrosis-factor (TNF)-alpha inhibitors such as adalimumab and infliximab have significantly improved CD management, especially in patients who have failed conventional treatments (e.g. steroids, 5-aminosalicylic acid, and immunomodulators) [20]. However, approximately one-third of patients fail anti-TNF treatment [21]. Monoclonal antibodies, for instance biologic agent vedolizumab, have been shown to be effective in TNF-alpha inhibitor non-responders, patients with contraindications to TNF-alpha inhibitors, and in patients who lose response or develop intolerance to such agents [22]. The human monoclonal antibody ustekinumab is a novel treatment option in CD which blocks the pro-inflammatory cytokines interleukin (IL)-12 and IL-23. The safety and efficacy of ustekinumab as an induction and maintenance therapy in moderate to severe CD has been evaluated and established in three phase 3 trials (UNITI-1 [23], n = 741; UNITI-2 [24], n = 628; and IM-UNITI [24, 25], n = 388). In the induction trials (UNITI-1 and UNITI-2), ustekinumab was found to produce significantly higher clinical response and remission rates compared to placebo both in patients who had failed conventional therapy and patients who had failed treatment with TNF-alpha inhibitors [23, 24]. Ustekinumab has been approved in the European Union for the treatment of moderately to severely active CD in patients who have an inadequate response with, or have lost response to, or are intolerant to either conventional therapy or a TNF-alpha inhibitor, or have medical contraindications to such therapies [26].

The objective of this study was to assess the cost-effectiveness of ustekinumab as a treatment of moderate to severe Crohn’s disease in Sweden. The cost-effectiveness of ustekinumab was investigated in two populations versus the relevant comparator in each population. TNF-alpha inhibitor adalimumab was the comparator in patients who had failed conventional care, some of which had been exposed to TNF inhibitor treatment without experiencing treatment failure or unacceptable side effects (“conventional care failure” population). The monoclonal antibody vedolizumab was the comparator in a population of patients who previously failed TNF inhibitor treatment (“TNF-alpha inhibitor failure” population).

Previous cost-effectiveness studies of ustekinumab in Crohn’s have been undertaken in British [27] and Polish [28] settings. Considering that cost-effectiveness analyses are highly contingent on local specifics such as cost and utility data, incidence and prevalence, as well as the health care system and clinical practice, the previous ustekinumab analyses are insufficient to draw conclusions for a Swedish context. Scandinavia sees the highest incidence and prevalence rates of Crohn’s in Europe [11], which highlight the value of evaluations of novel treatment options. The adaptation of the model to a Swedish context included adding productivity costs. A societal perspective captures the indirect costs of CD, which in addition to constituting a substantial proportion of the total costs, reflect the severe impact on Crohn’s patients’ everyday life.

This analysis aims to add to the understanding of the treatment possibilities and to enable future comparisons in a population with a considerable unmet need.

## Methods

### Model description

**Fig. 1:**
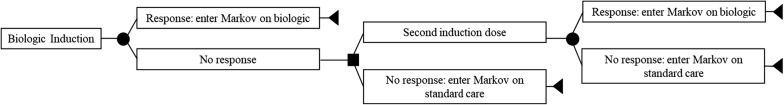
Induction phase decision tree

**Fig. 2:**
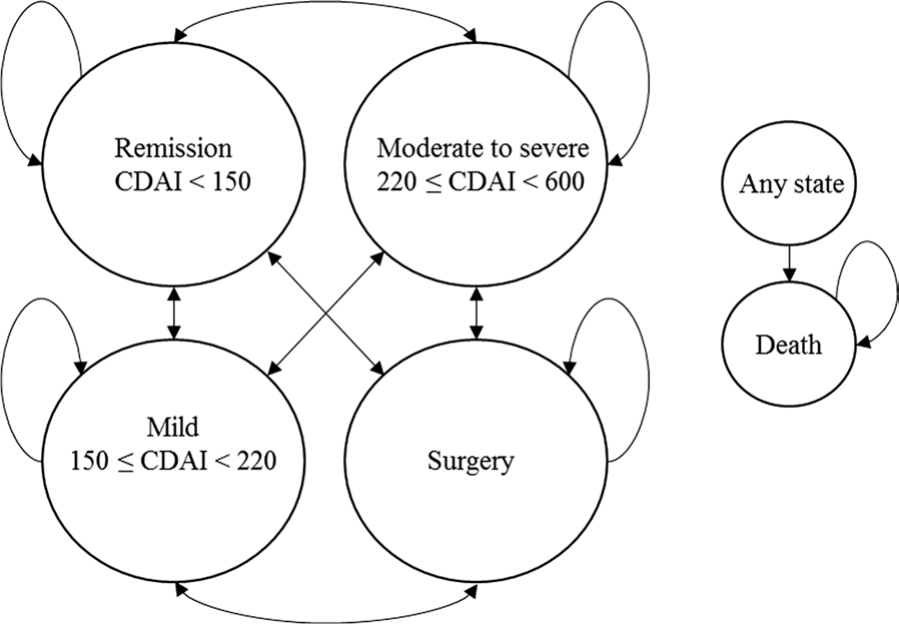
Maintenance phase Markov model. *CDAI* Crohn’s Disease Activity Index

The analysis was undertaken using a cost-effectiveness model based on previous work by Bodger et al. [20]. The original ustekinumab model was submitted to the National Institute for Health and Care Excellence (NICE) in 2016 [29], additional information is available in the evidence review group (ERG) publication [27] and report [30]. The model consists of a short-term induction phase, represented by a decision tree (Fig. 1) and a long-term maintenance phase, represented by a Markov cohort structure (Fig. 2). The model is structured around five health states, whereof three are based on disease severity measured by Crohn’s Disease Activity Index (CDAI) score [20, 31, 32] as follows:

- Moderate to severe CD: 220 ≤ CDAI < 600
- Mild CD: 150 ≤ CDAI < 220
- Remission: CDAI < 150

The two additional health states are surgery and death. The surgery state consists of patients who receive surgical treatment for their CD. Death may occur from any health state. The cycle length used was 2 weeks, and the model had a life time horizon (60 years) in line with Bodger et al. [20].

All patients enter the model in the moderate to severe health state. The patients initiate biologic induction therapy (Table 1), after which responders move to biologic maintenance treatment. Non-responders following the initial induction dose move either to receive a second induction treatment dose, or to non-biologic standard of care, defined as a combination of corticosteroids and immunosuppressants. Details of the initial and second induction doses are presented in Table 1. In the base case analysis, response was defined as a decrease in CDAI score of at least 100 points. The patients who respond to the second induction dose move to biologic maintenance treatment, whereas non-responders move to standard of care.

**Table 1:** Induction and maintenance doses

	First induction dose	Second induction dose	Maintenance dose
Ustekinumab	Weight based (IV): < 55 kg: 260 mg week 0 > 55 and < 85 kg: 390 mg week 0 > 85 kg: 520 mg week 0, response assessed at week 6	Additional 90 mg dose week 8, response assessed week 16 (SC)	90 mg every 12 weeks (SC)
Adalimumab	160 mg at week 0, 80 mg at week 2 (SC), response assessed at week 4	40 mg dose through week 12 (SC)	40 mg every 2 weeks (SC)
Vedolizumab	300 mg at weeks 0, 2 and 6 (IV), response assessed at week 10	Additional dose week 10, response assessed week 14 (IV)	300 mg every 8 weeks (IV)

*IV* intravenous, *SC* subcutaneous

The duration of the induction is 8 weeks for ustekinumab, 4 weeks for adalimumab and 10 weeks for vedolizumab.

In the maintenance phase, patients move between the health states depending on their level of treatment response in the induction phase and according to treatment transition probabilities.

### Treatments, efficacy and transition probabilities

The analysis compared ustekinumab to adalimumab in a conventional care failure population, and to vedolizumab in a TNF-alpha inhibitor failure population. Adalimumab is indicated in Sweden for moderate to severely active CD in adult patients who failed to respond despite complete and adequate treatment with corticosteroids and/or immunosuppressive therapy, or who are intolerant or have medical contraindications to such treatment [33]. Vedolizumab is indicated in Sweden for adult patients with moderate to severe CD who did not respond satisfactorily, demonstrated a decreasing response or were intolerant to conventional therapy or a TNF-alpha inhibitor [34].

The population relevant for ustekinumab in Swedish clinical practice would likely be patients with no initial response (primary non-response) or subsequent loss of response (secondary non-response) to a first line TNF-alpha inhibitor. The low price following the introduction of biosimilars in practice made infliximab the first TNF-alpha inhibitor option for CD in Sweden. At primary non-response to infliximab, many patients would receive vedolizumab, as it has a different mode of action. At secondary non-response, many patients would receive another TNF-alpha inhibitor such as adalimumab, with the purpose of regaining the initial response [35].

As there are no clinical trials with head-to-head evaluations of biologic treatments in moderate to severe CD, an indirect comparison was undertaken of randomized clinical trials (RCTs) of all comparators of interest. A systematic literature review in accordance with NICE guidelines was conducted, identifying all relevant studies on clinical data [29]. 41 publications reporting results from 31 different RCTs met the inclusion criteria. Network meta-analyses (NMAs) of short-term efficacy (probabilities of response or remission) for the induction phase were conducted for each population using a Bayesian framework [36]. As the identified maintenance trials were deemed not comparable for an NMA, comparative efficacy for the maintenance phase was estimated by means of a treatment sequence analysis, considering both induction and maintenance data. The objective of the NMA was to evaluate the relative efficacy of ustekinumab in moderate to severe CD in the previously discussed populations versus adalimumab and vedolizumab. The NMA conducted separate analyses for CDAI 70, 100 and 150. Induction phase trials were found similar enough to be pooled in a standard NMA, whereas the maintenance data alone was found non-comparable due to statistical heterogeneity and as the placebo arms differed too much. Maintenance efficacy is conditional on induction phase efficacy, and therefore the full treatment pathway needed to be considered, as the placebo effect sizes from the induction trials were deemed considerable. To account for treatment history, the treatment sequence analysis included induction and maintenance data for each of the interventions. The probability of responding at the end of the induction was multiplied with the conditional probability of maintaining response until the end of maintenance.

Similar assessment times in the induction trials were selected for each intervention in line with the primary endpoints of each trial included in the analysis. The NMA used a Bayesian hierarchical model, which preserved the randomization of each trial. Relative goodness of fit of the developed fixed- and random-effects models was assessed using the deviance information criterion (DIC). To achieve data driven results, in the base case analysis, non-informative prior distributions were used for unknown parameters (normal distributions with mean 0 and variance of 10,000 for treatment effects and uniform distributions for the between-trial standard deviation with range [0, 2] for binary outcomes). The NMA was performed in WinBugs using the Markov Chain Monte Carlo (MCMC) simulation method [37].

The inclusion and exclusion criteria in the identified vedolizumab [38, 39] and ustekinumab [23, 24] induction trials reflected the entire TNF-alpha inhibitor failure population, as they included both primary (initial non-response) and secondary (subsequent loss of response) non-responders to TNF-alpha inhibitors. Conversely, of the included adalimumab trials [40–42], one excluded TNF-alpha inhibitor primary non-responders [41], one included patients who had failed or were intolerant to one TNF-alpha inhibitor (infliximab) [42], and one excluded patients previously exposed to TNF-alpha inhibitors [40]. In accordance with the ustekinumab trials, and based on the available data for the comparators, vedolizumab was chosen as the comparator in a TNF-alpha inhibitor failure population, and adalimumab was chosen as the comparator in a conventional care failure population.

The only infliximab induction study identified as plausible for the comparison based on the inclusion criteria was a 20 year old, relatively small (n = 108) phase-II study [43]. There was a high level of missing data in the placebo arm (12%) classed as non-response, and the study observed an inverse dose response, i.e. higher effect in the lower infliximab doses. In addition, the magnitude of the observed results was never repeated in subsequent studies [29]. As this resulted in significant uncertainty in the indirect comparison it was decided not to include infliximab as a comparator.

Induction efficacy data for ustekinumab were sourced from the two induction trials UNITI-1 [23] and UNITI-2 [24], and induction efficacy for the comparators was based on the NMA. Induction probabilities of response and remission for adalimumab and vedolizumab were derived by applying odds ratios (ORs) calculated in the NMA to ustekinumab induction results. Induction transition probabilities were calculated from the rate of remission (α), the rate of response (β), and the percentage of responders who remain in the moderate to severe state (γ) for the respective state following Equation 1 (induction transition probabilities).

1 $$\begin{array}{*{20}ll} {Remission} {:\alpha} \\ {{Mild} {:\beta} \, - \,\left( {\alpha \, + \,\left( {\beta \,*\,\gamma } \right)} \right)} \\ {Moderate \, to \, severe}{:\beta \,*\,\gamma } \\ {{Non{\text{-}}responders} {:1}\, - \,\beta } \\ \end{array}$$

The rates of response and remission for each comparator and population are presented in Table 2. The percentage of responders who remain in the moderate to severe state (4.7% in the conventional care failure population and 6.0% in the TNF-alpha inhibitor failure population) was sourced from the ustekinumab maintenance trial [24, 25], and assumed to be the same for all treatments. The induction rate of surgery was assumed to be 2.0% [44]. Efficacy rates for the responders to the second induction dose were sourced from the ustekinumab [25] and vedolizumab [45] clinical trials, whereas the adalimumab rates were sourced from two efficacy studies [46] (Table 2).

**Table 2:** Induction response and remission rates (%)

	First induction dose		Second induction dose	
	Response	Remission	Response	Remission
Conventional care failure				
Ustekinumab	55.5	34.9	64.9	44.9
Adalimumab	54.8	45.6	43.0	28.0
TNF-alpha inhibitor failure				
Ustekinumab	33.7	18.5	41.1	18.4
Vedolizumab	32.7	12.9	16.0	6.8

*TNF* tumour necrosis factor

The maintenance efficacy for all treatments was based on the treatment sequence analysis. The distribution of patients across the health states at the beginning of the maintenance phase was defined using the proportion of patients in remission and response at the end of the induction phase. Once the starting distribution was completed, a transition matrix for transitions between the CDAI health states (remission, mild and moderate to severe) was applied for each treatment, and for each cycle until the end of the treatment duration. The transition matrix was calculated using Microsoft Excel tool Solver which derives transition probabilities to minimise Eq. 2 below, i.e. the difference between the calculated distribution of patients at the end of the maintenance phase, and the distribution predicted by the NMA.

2 $$\begin{aligned} & \left( {\%\;remission\;predicted\;by\;transition\;probabilities - \%\;remission\;predicted\;by\; NMA} \right)^{2} \\ & \quad + \left( {\% \;mild\;predicted\;by\; transition\;probabilities - \%\;mild\;predicted\;by\;NMA} \right)^{2} \\ \end{aligned}$$

Patients may discontinue treatment due to lack of efficacy with treatment specific cycle probabilities derived from clinical trials; ustekinumab data is sourced from IM-UNITI [24, 25], adalimumab data is sourced from infliximab trial ACCENT I [47] due to lack of available data and vedolizumab data is sourced from GEMINI II [39]. The cycle probabilities of discontinuation were applied to the proportion of patients in the moderate to severe health state, as it was assumed that this was the state patients would be in if there was a lack of efficacy. The percentage discontinued were as follows: ustekinumab: 10.6% (standard dose), 11.4% (escalated dose), adalimumab: 8.1%, vedolizumab 37.7% (standard dose) and 31.2% (escalated dose). The model used an annual rate of surgery of 7.0% and post-surgery transitions were sourced from a previous Markov cost-effectiveness analysis of biological therapy in CD [20].

Tables 1 and 2 presents the induction and maintenance dosages of all comparators and Table 3 contains the population specific patient characteristics. Subcutaneous injections (SCs) were assumed to be administered in-hospital (with a corresponding administration cost) during the induction phase, and at home by patients themselves (at no administration cost) in the maintenance phase. Intravenous infusions (IVs) were assumed to always take place in-hospital. The base case allowed dose escalation due to loss of response throughout the duration of treatment, with cycle probabilities of dose escalation of 2.0% for ustekinumab and vedolizumab [23] and 3.0% for adalimumab [48].

**Table 3:** Population specific patient characteristics

	Conventional care failure [49]	TNF-alpha inhibitor failure [23]
Mean age (years)	39.2	37.3
Mean weight (kg)	73.4	69.8
Proportion female (%)	52.9	57.2
Patients < 55 kg (%)	19.4	22.9
Patients 56–84 kg (%)	59.7	58.8
Patients > 85 kg (%)	20.9	18.4

*TNF* tumour necrosis factor, *kg* kilogram

50% of the patients on biologic treatment were assumed to receive standard of care in addition to their biologic treatment. In the base case analysis, all patients switched to standard of care treatment after 2 years, on which they remained for the duration of the simulation or until death. To reflect that post-treatment efficacy declines gradually, the transition matrices converged from biologic to standard of care transition probabilities over a treatment specific period of time based on the respective trials [24, 39, 48].

Health state specific utilities were sourced from the ustekinumab induction and maintenance trials [23–25] and mapped from inflammatory bowel disease questionnaire (IBDQ) score into EQ-5D values [50]. The resulting utilities were 0.80 in the remission state, 0.68 in the mild state and 0.55 in the moderate to severe state. The surgery state utility was assumed equal to the moderate to severe utility weight. The adverse events included were chosen based on expert opinion and the clinical trials of the comparators [23, 24, 39–42, 49]. Adverse events and the associated probabilities and disutilities are presented in Table 4.

**Table 4:** Utility decrements and cycle rates of adverse events

	Utility decrements	Cycle rates (%)		
		Ustekinumab [23–25]	Adalimumab [40, 41, 48, 51, 52]	Vedolizumab [39, 53]
Serious infection	− 0.52 [54]	0.34	0.32	0.32
Tuberculosis	− 0.55 [55]	0.00	0.00	0.00
Lymphoma	− 0.20 [56]	0.00	0.00	0.00
Hypersensitivity	− 0.11 [57]	0.01	0.00	0.00
Skin reactions	− 0.03 [58]	0.75	10.37	0.59

### Local cost data

For the purpose of this manuscript, costs have been converted from Swedish kronor (SEK) to euro (€) using the average 2016 European Central Bank (ECB) exchange rate (0.1057) [59]. All costs are presented in 2016 €.

The analysis employed a societal perspective, thus both direct and indirect costs were considered. Direct costs included drug costs, resource use in terms of in- and outpatient care and diagnostics/imaging, treatment administration, adverse events and surgical complication costs. The drug prices were sourced from the Swedish Dental and Pharmaceutical Benefits Agency (TLV) [60]. The unit cost of ustekinumab 90 mg was €3546, adalimumab 40 mg €533 and vedolizumab 300 mg €2334. An IV administration cost of €257 was sourced from a regional price list [61] and applied every time a dose was intravenously administered. In-hospital SCs had an associated administration cost of €124 [61]. Each administration occasion, regardless of dose counted as one administration in terms of costs.

Resource use costs and indirect costs in terms of productivity losses by health state were sourced from a Swedish cost study of CD [3] and inflated to 2016 values using consumer price index from Statistics Sweden [62]. Unit costs of adverse events were sourced from the regional price list [61]. The cost per surgical complication was based on the cost of additional in-patient days and outpatient attendances [61] associated with each respective complication. The expected number of additional days and attendances were sourced from the vedolizumab UK reimbursement submission [63] and validated by a Swedish clinical expert in gastroenterology. The resulting 2-week direct and indirect costs per health state are presented in Table 5.

**Table 5:** Local cost inputs

	Remission	Mild	Moderate to severe	Surgery
2-week cost of resource use	€26	€69	€108	€7123
2-week productivity cost	€79	€281	€374	€1326

### Cost-effectiveness analysis and uncertainties

The base case analysis compared ustekinumab to adalimumab in a conventional care failure population and to vedolizumab in a TNF-alpha inhibitor failure population. Costs and effects in terms of quality adjusted life-years (QALYs) were discounted at 3.0%, in line with Swedish health technology assessment (HTA) guidelines [64]. To understand uncertainty, a probabilistic sensitivity analysis (PSA), one-way sensitivity analyses and analyses of structural changes were performed. The one-way sensitivity analyses tested the model outcome’s sensitivity to changes in various inputs, and to changes in, for example, the time horizon and treatment duration. The structural changes tested variations in assumptions on dose escalation, health state costs, response criteria, and indirect costs. The cost-effectiveness outcome is presented using the incremental cost-effectiveness ratio (ICER).

## Results

### Base case analysis

#### Conventional care failure population

As shown in Table 6, a patient treated with ustekinumab was expected to accrue 14.275 QALYs at a cost of €232,225 over a life-time time-horizon (60 years). The corresponding for adalimumab was 14.043 QALYs gained, at a total cost of €239,209. Thus, ustekinumab had €6984 lower costs and a QALY gain of 0.232 as compared to adalimumab in the conventional care failure population. Indirect costs were the largest cost component for both treatments, consisting of approximately 50% of total costs.

**Table 6:** Disaggregated base case results including cost components and incremental results

Conventional care failure population					
	Cost components (€)				
	Drug costs (€)	Administration costs	Health state costs	Indirect costs	Adverse event cost
Ustekinumab	50,352	312	55,127	111,987	14,447
Adalimumab	41,942	996	57,767	118,962	19,543

	Total and incremental results				
	Cost (€)	Incremental cost	QALYs	Incremental QALYs	ICER (€)
Ustekinumab	232,225		14.275		
Adalimumab	239,209	6984	14.043	− 0.232	Dominated

TNF-alpha failure population					
	Cost components (€)				
	Drug costs (€)	Administration costs	Health state costs	Indirect costs	Adverse event cost
Vedolizumab	31,885	2293	62,767	132,895	14,881
Ustekinumab	43,501	336	61,206	128,843	14,859

	Total and incremental results				
	Total cost (€)	Incremental cost	Total QALYs	Incremental QALYs	ICER (€)
Vedolizumab	244,721		14.047		
Ustekinumab	248,745	4023	14.180	0.133	30,282

*QALY* quality adjusted life year, *ICER* incremental cost-effectiveness ratio, *TNF* tumour necrosis factor

#### TNF-alpha inhibitor failure population

As presented in Table 6, over a life-time time-horizon a patient treated with ustekinumab was expected to accrue 14.180 QALYs at a cost of €248,745. The corresponding for vedolizumab were 14.047 QALYs gained, at a total cost of €244,721. Thus, ustekinumab had a QALY gain of 0.133 as compared to vedolizumab, at a cost per QALY of €30,282 in the TNF-alpha inhibitor failure population. Again, the largest cost component for both arms was indirect costs, consisting of more than 50% of the total.

### Sensitivity analyses

Table 7 provides results of one-way sensitivity analyses as well as analyses of structural changes. The results were most sensitive to an increase of the treatment duration to 5 years. An increased treatment duration improved ustekinumab’s cost-effectiveness versus adalimumab but increased the ICER versus vedolizumab. The exclusion of adverse events had a relatively large impact in the conventional care failure population versus adalimumab. As it was suggested by a Swedish gastroenterologist that the cost of resource use in the moderate to severe health state was not sufficiently high, a sensitivity analysis was undertaken where the cost was doubled in the health state, which improved the cost-effectiveness of ustekinumab in both populations. Other structural changes analysed were CDAI-70 as response criteria, assuming no dose escalation, assuming no effect of adverse events and using different utilities, from an alternative source [20]. These sensitivity analyses had limited impact on the results in both populations.

**Table 7:** Sensitivity analyses

	Model input		ICER	
	Base case	Sensitivity	Ustekinumab versus adalimumab	Ustekinumab versus vedolizumab
Base case			Dominating	€30,282
*One-way sensitivity analyses*				
Discount rate cost	3%	0%	Dominating	€24,463
		5%	Dominating	€33,145
Discount rate health effects	3%	0%	Dominating	€25,946
		5%	Dominating	€33,091
Time horizon	60 years	15 years	Dominating	€33,707
		5 years	Dominating	€71,727
Treatment duration	2 years	5 years	Dominating	€104,952
*Analyses of structural changes*				
Indirect costs	Included	Not included	Dominating	€60,779
Discount rate cost and health effect	3%	0%	Dominating	€20,961
		5%	Dominating	€36,220
Dose escalation	Included	Not included	Dominating	€36,229
Resource use cost in moderate to severe health state		Doubled from base case	Dominating	€18,876
Response criteria	CDAI 100	CDAI 70	Dominating	€38,376
Effect of adverse events	Included	Not included	Dominating	€30,311
Utilities	IBDQ to EQ-5D	Bodger et al. [20]	Dominating	€27,980
		SF-36 to EQ-5D	Dominating	€63,188
		CDAI to EQ-5D	Dominating	€26,955

*ICER* incremental cost-effectiveness ratio, *CDAI* Crohn’s Disease Activity Index, *IBDQ* inflammatory bowel disease questionnaire, *EQ-5D* EuroQol-5D

**Fig. 3:**
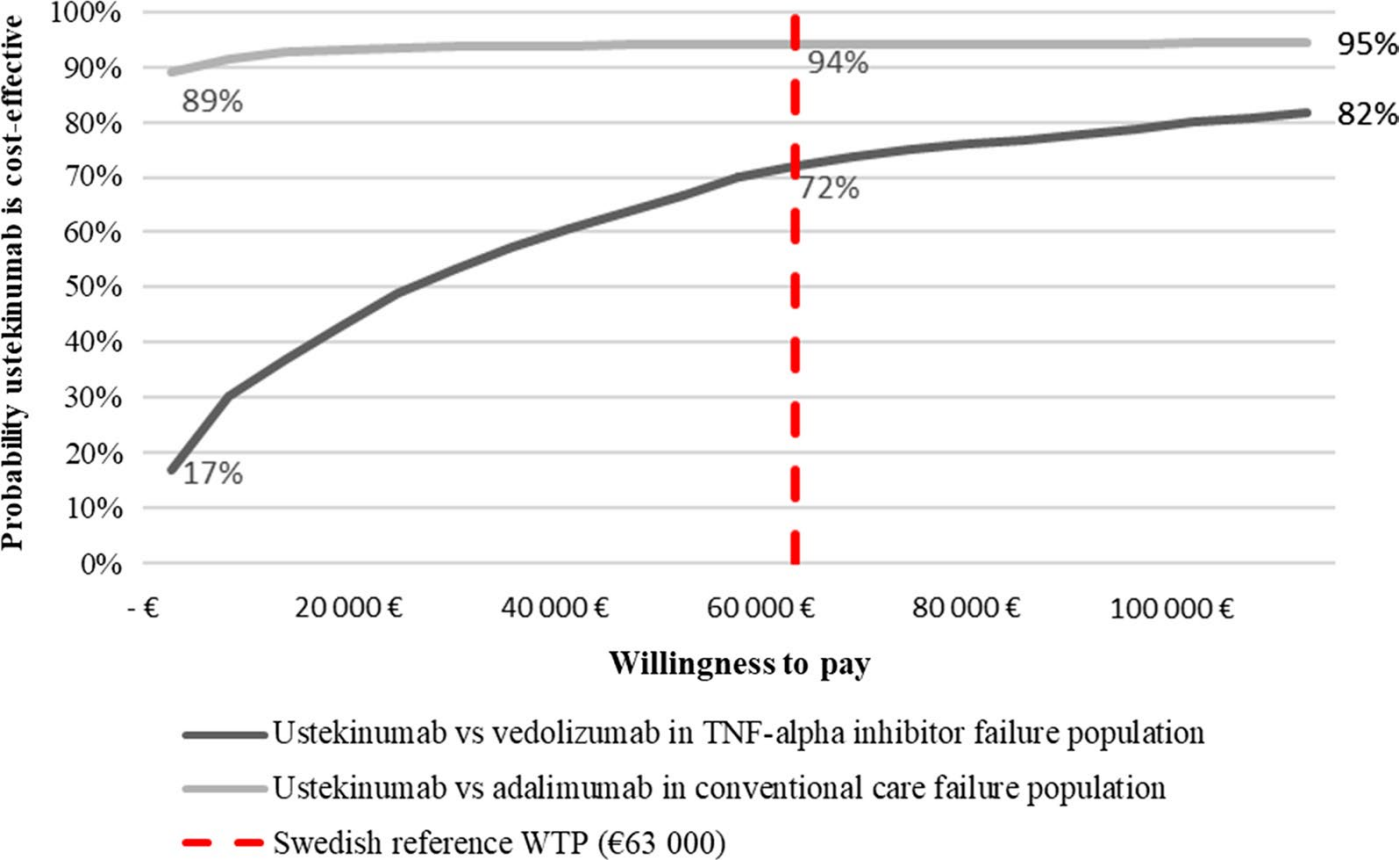
Cost-effectiveness acceptability curves. *TNF* tumour necrosis factor, *WTP* willingness to pay

To test the robustness of the results with respect to uncertainty in the model input parameters, a PSA was performed for both populations, using 1000 iterations. The cost effectiveness acceptability curves (Fig. 3) suggest that ustekinumab has a 94% probability of being cost-effective compared to adalimumab and 72% versus vedolizumab at a willingness to pay (WTP) of €63,000 (SEK 600,000) in the respective populations. This WTP level was chosen as it is commonly referred to as a reference WTP in Sweden [65].

## Discussion

Assuming a lifetime time horizon and 2-years treatment duration, the results indicate that ustekinumab dominates adalimumab in the conventional care failure population with a QALY gain of 0.232, and is cost-effective compared to vedolizumab in the TNF-alpha inhibitor failure population. The QALY gain versus vedolizumab was 0.133 and the cost per QALY was €30,282, which is below the Swedish reference WTP. The results were robust to several univariate sensitivity analyses, and the PSA. Ustekinumab was dominating adalimumab in all sensitivity analyses. Considering the Swedish reference WTP, ustekinumab was cost-effective versus vedolizumab in all one-way sensitivity analyses, with the exception of when the treatment duration was increased to 5 years and when the time horizon was decreased to 5 years. Besides treatment duration and time horizon, using different utilities and the exclusion of indirect costs had the largest impact on the results.

To our knowledge, there are no other cost-effectiveness studies of ustekinumab in moderate to severe CD in Sweden. Therefore, it is not straightforward to make a comparison to previous literature. There are no head-to-head trials between biologic agents, and there is a great variation in assumptions and data sources used by cost-effectiveness studies of biologics in CD [66]. This results in considerable variations in the outcomes of the studies. For example, a review of the cost-effectiveness of biologics in CD patients found 12 such studies, with ICERs versus conventional care ranging from dominating to €549,335 [67]. Another study assessed the cost-effectiveness of ustekinumab versus vedolizumab from a payer perspective in the US, in a population of TNF-alpha inhibitor naïve, non-TNF-alpha inhibitor refractory patients. Considering a lifetime perspective, vedolizumab accrued 10.461 QALYs whereas ustekinumab accrued 10.326 with incremental costs of $55,523 in favour of vedolizumab [68]. This comparison was done in a different population than ours, and little information on the assumptions was presented. A straightforward comparison on the difference to our outcome is therefore impossible. Another analysis of ustekinumab’s cost-effectiveness versus vedolizumab as a third line agent after failure with two TNF-alpha inhibitors found that vedolizumab had higher costs of $168,648 as well as a larger QALY gain of 0.029, resulting in an ICER of $5,815,448 [69]. A polish study found treatment with ustekinumab after failing one TNF-antagonist associated to a QALY gain of 0.349 and an ICER of €18,878 compared to adalimumab after infliximab failure [28]. The variations in outcomes of cost-effectiveness analyses of biologics in CD emphasise the importance of further research in the field, and a need for a greater consensus over model design and assumptions.

Similar model structures as the one utilized for this study have previously been used in HTAs of biological CD treatments [29, 63, 70, 71]. The analysis is based on the best available clinical evidence and publicly available data. The ustekinumab induction trials (UNITI-1 and UNITI-2) had high internal validity. As a means of improving flexibility, the model allowed continued induction response assessment. As there are no head-to-head trials between biologics in CD, efficacy between the comparators had to be indirectly estimated. NMAs were performed for the induction phase and treatment sequence analyses were utilised for the maintenance phase for the respective populations. The clinical endpoints in the induction trials were estimated at week 6 for ustekinumab and vedolizumab, and week 4 for adalimumab. The efficacy assessment time points in the NMA were in line with the included studies [29].

Previous failure to TNF-alpha inhibitor treatment was found to impact the results of the NMA. Therefore, separate analyses were undertaken between trials including conventional care failure patients and TNF-alpha inhibitor failure patients. Moreover, a sensitivity analysis was undertaken in which conventional care failure inputs were replaced with inputs for patients truly naïve to biologics, providing evidence of robustness to the results. To further validate the results of the NMA, and to investigate potential sources of bias, several other sensitivity analyses were undertaken which supported the base case outcome [29].

A common issue in performing indirect comparisons is heterogeneity between the source trials. The placebo arms were not comparable across the maintenance trials, and could therefore not be used as a common comparator to anchor the indirect comparison, rendering a traditional NMA impossible. Moreover, statistical analyses revealed significant levels of heterogeneity. As often in maintenance trials, patient selection was based on induction response to the evaluated treatment. Additionally, the patient profiles in terms of previous TNF-alpha inhibitor treatment failure differed over the trials. The adalimumab trials only included secondary non-responders, whereas the vedolizumab and ustekinumab trials included primary non-responders as well. As the latter inclusion criteria allowed patients with more severe disease, the effect of ustekinumab and vedolizumab risked being underestimated compared to adalimumab in an indirect comparison. As the maintenance effect is a product of initial treatment effect, the full treatment pathway had to be considered. A treatment sequence analysis was therefore the most plausible option to obtain long-term relative efficacy. Not only did this increase the comparability between maintenance trial placebo arms, it also allowed an evaluation of effect over the full treatment sequence [36].

The first cost-effectiveness model for CD was developed by Silverstein et al. [32] using data from an observational US cohort (1970–1993) from Olmstead County. It was published in 1999. More recently, in 2009, Bodger et al. [20] adapted the Silverstein model to a UK context. CD models have since then been developed for three NICE appraisals. Namely appraisals of infliximab and adalimumab [71], of vedolizumab [63], and of ustekinumab [29]; the latter being the model which this study is based on. The previous models’ limitations include not capturing the relapsing–remitting nature of CD, overly simplifying surgery by disregarding that subsequent surgery often is contingent to first surgery, and using too short time horizons to capture long-term outcomes. As CD is a chronic condition, such outcomes are important to understand the full implications of the disease.

The Bodger et al. model structure on which this model builds have some limitations. It does not allow for treatment sequencing or re-treatments. This is less critical in our analysis, as the populations evaluated are not treatment naïve, but have experienced either conventional care or TNF-alpha inhibitor treatment. Thus, we indirectly model treatment sequences, but with initial treatment taking place outside of the simulation, reflected in the patient data. Additionally, properly modelling treatment sequences would require currently unavailable efficacy data, or ill-founded assumptions. Although the model allows for maintenance transitions from the moderate to severe health state, the structure does not capture increased relapse risk after discontinuing biologics [28, 72, 73], and—as previously discussed—does not consider the impact of surgery on future surgeries.

In the development of the ustekinumab model, such limitations were considered. For example, in the model patients can transition from the moderate to severe health state in the maintenance phase, thus reflecting that CD is a relapsing–remitting disease. Developments of the surgery modelling was explored, but resulted in non-intuitive outcomes. Other model developments to overcome previous limitations included differentiating the induction length of the comparators, adding the possibility to test different treatment lengths, and—specifically based on input from three health economic experts and a leading clinician—including a gradual decline in post-treatment efficacy in the maintenance period.

The Solver approach to estimate transition probabilities have a number of limitations, as pointed out by the ERG [30]. The approach is contingent on constraints and starting values, which although based on a previous NICE submission [63], were criticized for increasing uncertainty while having a substantial impact on the transition probabilities and subsequently the cost-effectiveness. Previous criticism was however accounted for by the inclusion of additional calibration constraints.

To validate the model, a comparison was done between model outcomes and the predicted outcomes of the treatment sequence analysis. The comparison concerned 1-year outcomes of the proportion of patients ending up in each health state. The model outcomes were very close to the predicted outcomes—with a slight bias against ustekinumab in the conventional care failure population [29].

Patients who did not respond to the initial and second induction doses were assumed to remain in the moderate to severe health state and spend the remainder of the simulation on standard of care. This is a simplification of clinical practice, but was deemed the most reasonable assumption in absence of adequate data. Some inputs were unavailable for a Swedish setting, for instance no adverse event risk data in Swedish CD patients was identified. Therefore, it was assumed that the same risk as estimated in moderate to severe CD patients in the UK apply to Swedish patients. Modelling assumptions inevitably produce uncertainty, but in order to reduce it to the greatest extent possible, assumptions related to data gaps, model inputs, and treatment regimens were validated by Swedish clinical experts in gastroenterology.

As this study was designed to capture Swedish health economic circumstances, the generalisability of the conclusions to an international context is limited. However, the observed cost-effectiveness outcomes are likely similar within other regions with similar CD incidence and prevalence, and comparable health care systems such as for instance, the other Nordic countries.

## Conclusions

In conclusion, based on available evidence, and while acknowledging the inherent limitations of cost-effectiveness modelling; from a societal perspective, ustekinumab is dominating adalimumab in Sweden in patients who failed conventional treatment. Moreover, the results indicate that ustekinumab is likely to be cost-effective versus vedolizumab in patients who previously failed treatment with TNF-alpha inhibitors.

## Abbreviations

CD: Crohn’s disease
CDAI: Crohn’s Disease Activity Index
DIC: deviance information criterion
ECB: European Central Bank
ERG: evidence review group
HRQoL: health related quality of life
HTA: health technology assessment
IBDQ: inflammatory bowel disease questionnaire
ICER: incremental cost-effectiveness ratio
IL: interleukin
IV: intravenous
MCMC: Markov Chain Monte Carlo simulation method
NICE: National Institute for Health and Care Excellence
NMA: network meta-analysis
OR: odds ratio
PSA: probabilistic sensitivity analysis
QALY: quality adjusted life-years
RCT: randomized clinical trials
SC: subcutaneous
SEK: Swedish kronor
TLV: The Swedish Dental and Pharmaceutical Benefits Agency
TNF: tumour necrosis factor
WTP: willingness to pay
